# Equal access to outreach mental health care? Exploring how the place of residence influences the use of intensive home treatment in a rural catchment area in Germany

**DOI:** 10.1186/s12888-022-04477-y

**Published:** 2022-12-26

**Authors:** Julian Schwarz, Jan Hemmerling, Nadja Kabisch, Laura Galbusera, Martin Heinze, Sebastian von Peter, Jan Wolff

**Affiliations:** 1 University Clinic for Psychiatry and Psychotherapy, Brandenburg Medical School, Immanuel Hospital Rüdersdorf, Rüdersdorf, Germany; 2 Centre for Health Services Research, Brandenburg Medical School, Rüdersdorf, Germany; 3 Faculty for Health Sciences, Brandenburg Medical School, Neuruppin, Germany; 4 Geography Institute, Humboldt Universität zu Berlin, Berlin, Germany; 5 Institute for Physical Geography and Landscape Ecology, Leibniz University Hannover, Hannover, Germany; 6 Peter L. Reichertz Institute for Medical Informatics of the TU Braunschweig and the Medical University Hannover, Hannover, Germany

**Keywords:** Health care access, Health equity, Home-treatment, Crisis resolution teams, Outreach care, Health care planning, Geography, Spatial analysis, Mental health services, Stationsäquivalente Behandlung, StäB

## Abstract

**Background:**

Internationally, intensive psychiatric home treatment has been increasingly implemented as a community-based alternative to inpatient admission. Since 2018, the so-called Inpatient Equivalent Home Treatment (IEHT; German: "Stationsäquivalente Behandlung", short: "StäB") has been introduced as a particularly intensive form of home treatment that provides at least one daily treatment contact in the service users’ (SU) home environment. Prior research shows that this can be challenging in rural catchment areas. Our paper investigates to which extent the location of the SU home location within the catchment area as well as the distance between the home and the clinic influence the utilisation of inpatient treatment compared to IEHT.

**Method:**

Routine data of one psychiatric hospital in the federal state of Brandenburg in Germany were analysed for the observational period 07/2018–06/2021. Two comparison groups were formed: SU receiving inpatient treatment and SU receiving IEHT. The SU places of residence were respectively anonymised and converted into geo-coordinates. A geographic information system (GIS) was used to visualise the places of residence, and car travel distances as well as travel times to the clinic were determined. Spatial analyses were performed to show the differences between comparison groups. In a more in-depth analysis, the proximity of SU residences to each other was examined as an indicator of possible clustering.

**Results:**

During the observational period, the location of 687 inpatient and 140 IEHT unique SU were mapped using the GIS. SU receiving treatment resided predominantly within the catchment area, and this proportion was slightly higher for SU receiving IEHT than for those treated in inpatient setting (95.3% vs. 84.7%). In the catchment area, the geographical distribution of SU place of residence was similar in the two groups. There was a general higher service provision in the more densely populated communities close to Berlin. SU with residence in peripheral communities were mainly treated within the inpatient setting. The mean travel times and distances to the place of residence only differed minimally between the two groups of SU (*p* > 0.05). The places of residence of SU treated with IEHT were located in greater proximity to each other than those of SU treated in inpatient setting (*p* < 0.1).

**Conclusion:**

In especially peripheral parts of the examined catchment area, it may be more difficult to have access to IEHT rather than to inpatient services. The results raise questions regarding health equity and the planning of health care services and have important implications for the further development of intensive home treatment. Telehealth interventions such as blended-care approaches and an increase of flexibility in treatment intensity, e.g. eliminating the daily visit requirement, could ease the implementation of intensive home treatment especially in rural areas.

## Background

Equal access to care services is a leading principle of health care systems [1]. A central goal of these systems is providing medical services according to the needs of the population and as community-based as possible. The access to services is influenced by non-spatial (e.g. economic, cultural and social) as well as spatial factors, such as the local availability of health care infrastructures and the geographic distance to them.

As early as 1866, the epidemiologist and psychiatrist E. Jarvis was able to prove a significant correlation between the rate of admission and the distance of service users’ (SU) place of residence to the psychiatric hospital. SU that lived close to the hospital were more likely to be treated as inpatients than those that lived further away [2]. This so-called “Distance Decay” effect has been demonstrated in a variety of studies on psychiatric settings [1, 3–8]. In 2011, Zulian et al. showed that the citizens of a rural community in the region of Verona (Italy) were less likely to utilise a healthcare facility, the more the (car travel) distance separated their place of residence and the service provision [1]. Following the assumption that psychiatric SU often do not have a car, Stulz et al. were able to prove in a more recent study that the utilisation of outpatient psychiatric services recedes with increasing travel time by public transport [3]. This difference did not apply to inpatient services though, which were equally accessed by SU regardless of the distance between their home and the hospital [3].

To date, it has not been investigated to what extent the usage of (intensive) home treatment depends on the place of residence, or whether a greater distance between the place of residence and the hospital constitutes a negative predictor for outreach psychiatric service usage. Since outreach intensive care services are being internationally increasingly implemented as an alternative to inpatient psychiatric care [9–12], this issue is nowadays of great clinical and scientific relevance. In contrast to other settings, acute outreach mental health care enables a stronger integration of treatment into SU everyday life and social network (and vice versa) through mobile, multi-professional teams, and is recommended in several guidelines with the highest level of evidence [13, 14]. Consequently, access to inpatient-treatment-replacing care services should generally be ensured for all SU in the interests of equity of care.

In Germany, the possibility for psychiatric hospitals with a catchment area to offer the so-called Inpatient-Equivalent Home Treatment (IEHT; according to §115d social code V) as part of routine health care services exists since 2018-01-01 [15, 16]. The legal framework specifies that this intensive form of home treatment must be equivalent to inpatient psychiatric treatment in terms of service content, complexity and flexibility, and that thus a multi-professional team is required to perform at least one daily visit in the home environment. At the moment, about 50 hospitals within Germany with a mainly urban catchment area provide IEHT, with a rising tendency [17]. In rural areas the provision of IEHT is made difficult by longer travel distances and lack of personnel [18]. Especially the legally required daily treatment contacts present a challenge in the care of SU that live far away from the hospital.

Thus, the goal of this study is to investigate to what extent IEHT, a particular intensive form of home treatment, is utilised in a large rural catchment area in comparison to inpatient treatment, and whether this is influenced by the previously mentioned distance effects. The following research questions are explored:

1. Is intensive home treatment utilised equally across the catchment area studied, or are there differences depending on the place of residence?
2. How do the distances and the car travel time from place of residence to hospital differ between SU treated in the home treatment or inpatient setting?

## Method

### Design

Analyses of the utilisation and mapping of health care services have been performed for research and planning purposes using geographic information systems (GIS) [7, 19]. This study uses the „Google Distance Matrix “interface (Google LLC, California, USA) to depict the usage of IEHT and inpatient treatment within one hospital catchment area and to calculate the car travel times and distances between hospital and the SU home address. Furthermore, spatial analyses have been performed to demonstrate a possible “Distance Decay” effect and clustering as an indicator for an irregular distribution of SU treated within the catchment area [20–22]. The difference between both settings has been determined using descriptive statistics. The analysis is based on hospital routine data (according to §21 hospital remuneration law). The ethics committee of the Medical School Hannover confirmed that our study did not require ethical oversight. Anonymity and confidentiality were ensured in that only anonymous data without personal reference were processed.

### Setting

The study was performed at the Department for Psychiatry and Psychotherapy of the Brandenburg Medical University, Immanuel Hospital Rüdersdorf (IHR). Additionally, to the main site in Rüdersdorf, two satellite locations in Strausberg and Fürstenwalde belong to the hospital, and both include a day-care unit and a psychiatric outpatient centre. The IHR is responsible for the acute mental health care for the two regions Märkisch-Oderland (MOL) and Oder-Spree (LOS) that border Berlin to the east. The catchment area of the hospital includes 239.908 inhabitants (recorded 2020-12-30) and a surface of 1.550 km^2^. This area is just about double the size of Berlin. 70% of the inhabitants of the catchment area live in the more densely populated communities close to Berlin [23].

**Fig. 1:**
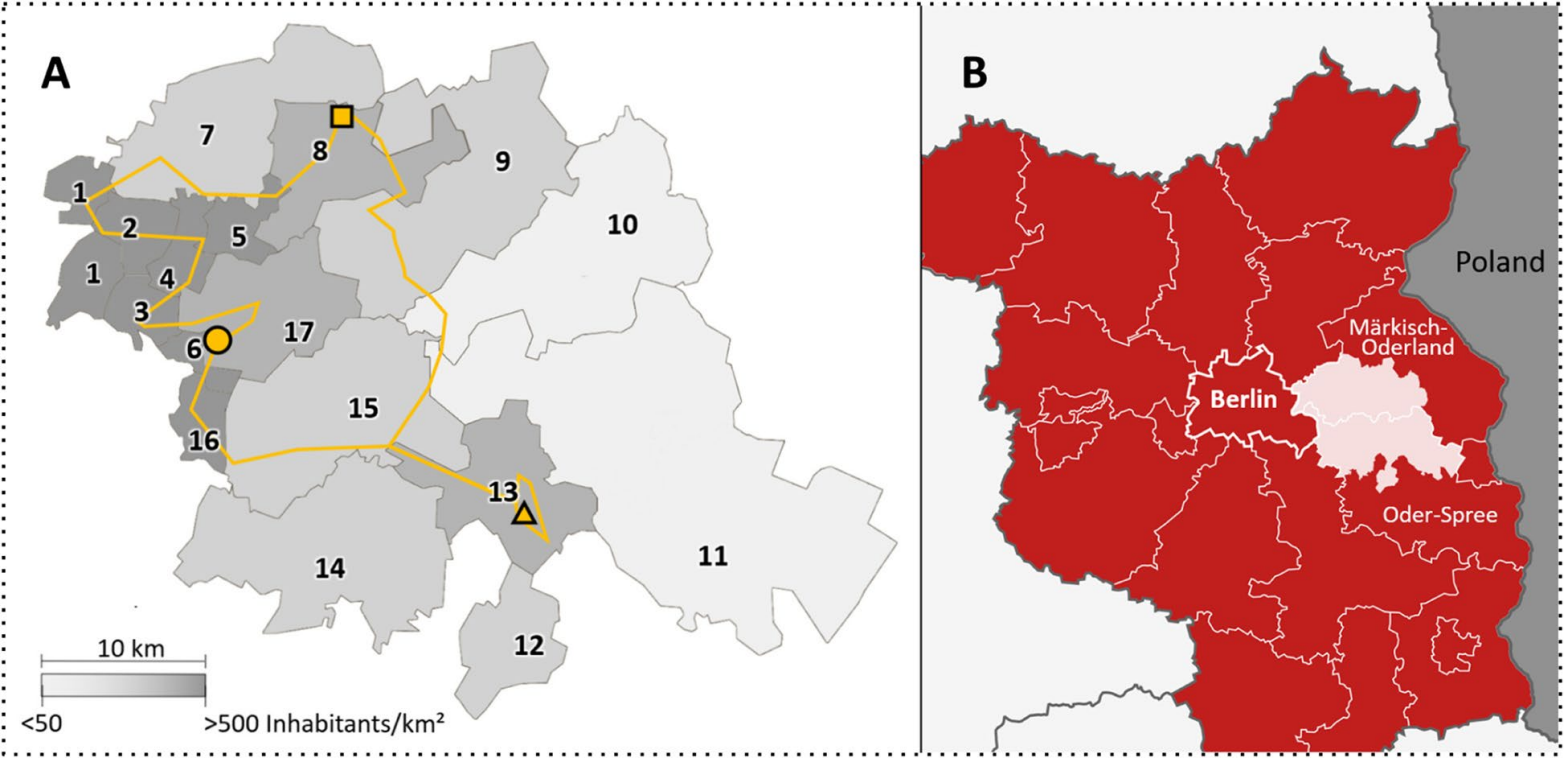
Catchment area of the study hospital (**A**) and its geographical position within the state of Brandenburg (**B**). **A:** The orange line marks exemplary travel routes of the IEHT team; the orange circle marks the position of the hospital (Rüdersdorf), the satellite units (each including a day-care centre and an outpatient centre) are represented by a square (Strausberg) and a triangle (Fürstenwalde). The numbers 1–17 mark the various communities, cities and administrative offices of the catchment area. The colouring of the regions in different shades of grey corresponds to the respective population density (see figure for legend). **B:** The catchment area of the study hospital (white) as part of the Brandenburg districts Märkisch-Oderland (MOL) and Oder-Spree (LOS). The other areas marked in red represent the districts of the states of Brandenburg and Berlin

At IHR, IEHT has been offered since May 2018. During the observational period (1. July 2018–30. June 2021) the mean caseload of IEHT, i.e. the amount of SU treated simultaneously, was 7.2. Treatment contacts were usually performed by two team members working together, driving by car from one SU to the next. Depending on the distribution of SU within the catchment area, one or two different routes, a north-western and a south-eastern one, were travelled every day. The mean length of an exemplary travel route (Fig. 1) amounted to roughly 126 km with a travel time of about 2 hours and 45 minutes. In principle, all SU within the entire catchment area can be treated in the IEHT setting as long as the criteria for inpatient hospital admission are met, i.e., an acute psychiatric disorder is present and the treatment goals are likely to be best achieved in the IEHT setting. In addition, the home environment must be suitable for the provision of IEHT. This is not the case, for example, if there is no privacy for a therapeutic one-on-one conversation, or child welfare risks are imminent or already exist. SU with a primarily substance-related disorder, for example, were predominantly treated in the inpatient setting in order to enable a safe qualified withdrawal and to prevent a relapse in the home environment.

### Data synthesis and analysis

All SU who were treated in the period of 07/01/2018–06/30/2021 in the inpatient or IEHT setting were identified using the unique patient ID from the routine data of the IHR. Patients from the Techniker and Barmer statutory health insurance companies - around 30% of the cases treated at the IHR - were not included in the sample, since they do not receive IEHT but a different form of outreach treatment [24, 25], which is not the subject of the present study. Two comparison groups were formed: one for SU receiving inpatient treatment and, the other for those receiving IEHT. If a SU was treated in both settings, one was assigned to both comparison groups. SU home addresses were exclusively extracted from the hospital information system and assigned to the respective comparison group without any specific connection to the patient ID number. The addresses were converted to geographical coordinates using the GIS “GoogleMaps API “and analysed with the software Rstudio (RStudio Inc., Boston, USA). SU with addresses that were either incomplete or could not be identified by the GIS were removed from the sample.

All places of residence were plotted on a map of the catchment area for a visual presentation. The car travel route between hospital and SU residence with the shortest possible travel time and distance was calculated for all SU. To test a possible „Distance Decay “ effect, the amount of SU treated per setting was set in relation to the increasing travel time. This was extended by a more differentiated presentation showing the distribution of the primary diagnoses in relation to the increasing travel time.

In order to obtain a comparable measurement for the geographical distribution and possible clustering, the mean paired distances (linear distances) between all residences per setting were calculated. Visual interpretation indicated differing spatial distribution especially with decreasing proximity of SU between settings. Therefore, in each setting, the 75% quantile of the paired distance was calculated for each of the residences and the distribution subsequently tested for significant differences. Thereby, particular differences in the pairwise distance outside of existing spatial clusters could be taken into account. All differences between comparison groups were tested using the Wilcoxon-Mann-Whitney-Test.

## Results

Within the observation period, 831 SU were treated in the inpatient and/or the IEHT setting by the study hospital. 783 addresses of this total number of SU could be determined with the GIS, and of those, 687 SU were treated in inpatient setting and 140 SU received IEHT. 44 SU received care in both inpatient and IEHT settings. The socio-demographic characteristics of the service users are shown in Table 1.

**Table 1:** Socio-demographic characteristics of the service users treated in the inpatient and inpatient-equivalent home treatment (IEHT) setting

Parameter	﻿Setting	
	Inpatient	IEHT
n	687	140
Female gender, n (%)	363 (52.9)	87 (62.3)
Age, M (SD)	50.49 (19.00)	54.32 (18.73)
LOS, M (SD)	25.70 (28.73)	31.32 (21.88)
Primary diagnosis		
F0	48	9
F1	81	5
F2	139	35
F3	305	68
F4	93	16
F5	2	0
F6	12	5
Other	6	1
Psychiatric comorbidity, M (SD)	1.03 (1.29)	0.75 (1.05)
Somatic comorbidity, M (SD)	3.16 (3.27)	2.10 (2.44)

*IEHT* Inpatient-equivalent home treatment, *LOS* Length of stay, *M* Mean, *SD* Standard deviation

### Descriptive comparison

**Fig. 2:**
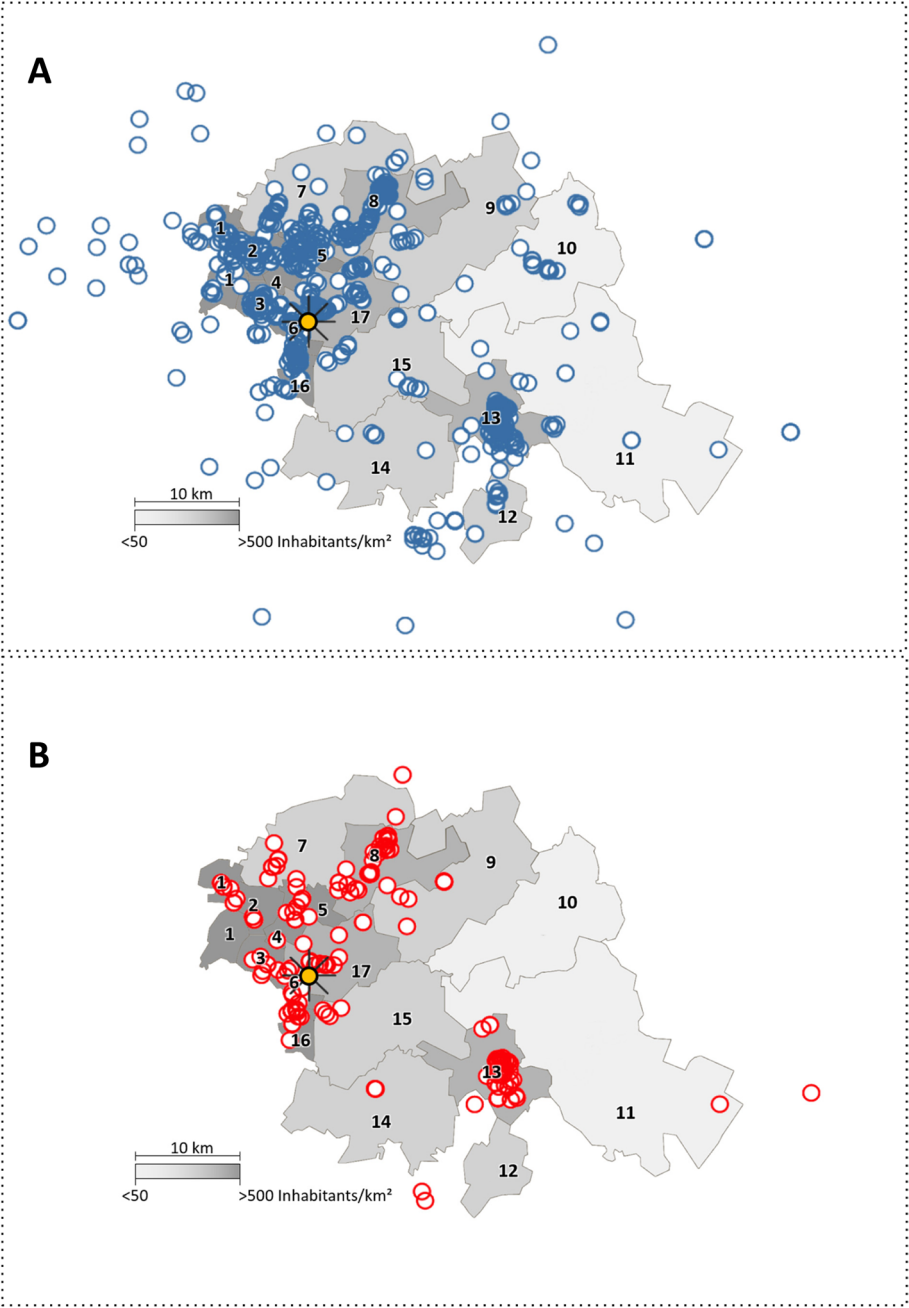
Distribution of service users’ locations in- and outside of the catchment area treated in the inpatient (**A**) and inpatient-equivalent home treatment (IEHT) (**B**) setting. The orange circle marks the position of the study hospital; the circles mark the locations of one or more service users (blue = inpatient; red = IEHT). The numbers 1–17 mark the communities and cities of the catchment area

Generally, a similar distribution between SU of the two treatment settings can be shown in the map (Fig. 2), with one difference: places of residence of SU who received inpatient treatment are scattered over a wider area whereas those of SU receiving IEHT are more clustered. The majority of SU who received treatment live in the proportionally more densely populated communities (with population density > 227/km^2^) and immediately adjacent surroundings (Table 2). Of these, about 2/3 live in cities and communities neighbouring Berlin (Fig. 2, Nr. 1–6, 8, 16) and 1/3 in the smaller city of Fürstenwalde in the southeast of the catchment area (Fig. 2, Nr. 13). In four communities that comprise in total 51.0% of the area and 15,2% of the population of the whole catchment area, SU were treated predominantly or even exclusively in the inpatient setting. These are the especially sparsely populated and peripheral communities (Table 2 and Fig. 2, Nr. 10–12 and 14).

**Table 2:** Percentage of regional surface, inhabitants, and service users treated in the inpatient and inpatient-equivalent home treatment (IEHT) setting in the communities of the catchment area

*City/ Community*	*Surface (%)*^*1*^	*Residents (%)*^*1*^	*Population density*^*2*^	*Inpatient setting (%)*^*3*^	*IEHT setting (%)*^*3*^
*1*	*2.10%*	*7.60%*	*569*	*6.01%*	*3,01%*
*2*	*1.30%*	*7.80%*	*960*	*5.84%*	*3.01%*
*3*	*1.10%*	*5.40%*	*771*	*5.50%*	*5.26%*
*4*	*1.10%*	*6.00%*	*871*	*6.19%*	*7.52%*
*5*	*1.10%*	*6.40%*	*877*	*4.81%*	*2.26%*
*6*	*0.60%*	*3.50%*	*924*	*2.75%*	*3.01%*
*7*	*6.90%*	*4.00%*	*91*	*4.81%*	*6.02%*
*8*	*4.40%*	*11.20%*	*397*	*13.40%*	*17.29%*
*9*	*12.20%*	*4.10%*	*52*	*2.58%*	*4.51%*
*10*	*9.80%*	*2.90%*	*46*	*2.41%*	*–*
*11*	*22.00%*	*4.30%*	*30*	*2.23%*	*0.75%*
*12*	*8.00%*	*4.30%*	*84*	*2.06%*	*–*
*13*	*4.60%*	*13.30%*	*453*	*19.76%*	*24.06%*
*14*	*11.20%*	*3.70%*	*51*	*1.20%*	*0.75%*
*15*	*8.20%*	*3.70%*	*70*	*2.75%*	*3.01%*
*16*	*1.10%*	*5.00%*	*722*	*6.70%*	*9.02%*
*17*	*4.50%*	*6.70%*	*228*	*11.00%*	*10.53%*

*IEHT* Inpatient-equivalent home treatment, *SU* service user, 1: In relation to the total surface/inhabitants of the catchment area, 2: Inhabitants per km^2^, 3: Share of SU treated in the respective setting during the observational period

### Spatial analyses

**Fig. 3:**
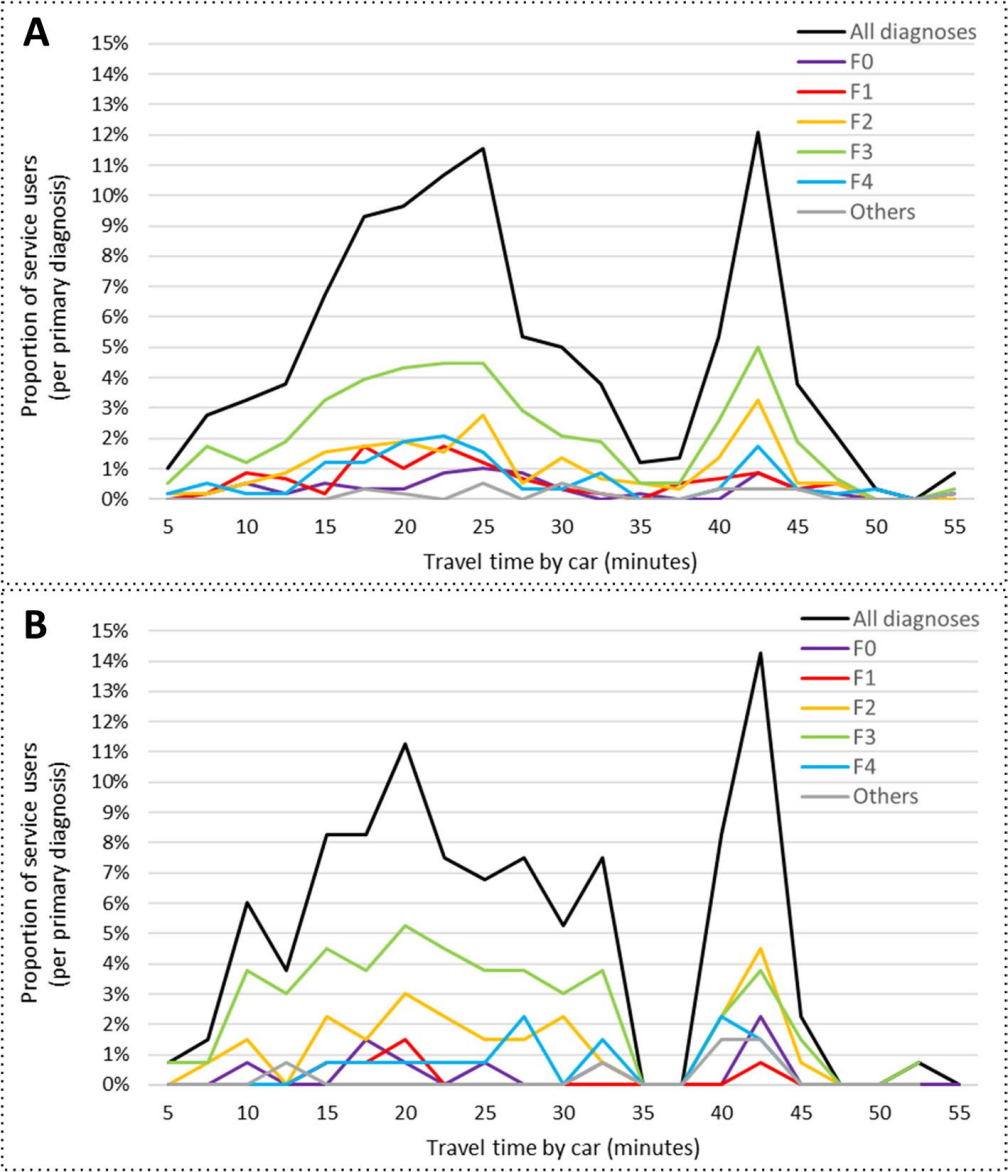
Car travel times for service users with different primary diagnosis treated in the inpatient (**A**) and inpatient-equivalent home treatment (IEHT) (**B**) setting within the catchment area. The percentages refer in each case to the share of the total number of service users treated within the inpatient (A; *n* = 582) or the IEHT (B; *n* = 133) setting. The peaks mark locations with an increased amount of served service users (e.g. 40-45 minutes travel time corresponds to the city of Fürstenwalde, No. 13 in Table 2)

In the following spatial analyses only SU residing inside the catchment area were included, comprising 84,7% (*n* = 582) of SU in the inpatient and 95.3% (*n* = 133) in the IEHT setting. Figure 3 shows that the number of SU in treatment varies with increasing travel time (and distance) from hospital to their place of residence rather than decreasing. This distribution seems to be basically similar for SU treated in the inpatient as well as in the IEHT setting, regardless of their primary diagnosis.

The mean travel times, distances and the paired distances between the SU places of residence (as an indicator for clustering) are displayed in Table 3. The differences between the two comparison groups are not statistically significant for travel time and distance. The analysis of the paired distances between places of residence shows that SU having received IEHT live on average closer to one another than within the inpatient comparison group (*p* = 0.0965). Considering the 75% quantile, this result is significant with *p* < 0.01.

**Table 3:** Travel times, travel distances and paired distances between places of residence for service users treated in the inpatient or inpatient-equivalent home treatment (IEHT) setting within the catchment area

Comparison Group:	﻿Setting		
	Inpatient	IEHT	MW-Test (p)
Number of SU; n	582	133	
Travel time in minutes; M (SD)	25.69 (11.42)	26.00 (11.48)	0.3911
Travel route in km; M (SD)	21.34 (14.93)	21.82 (14.93)	0.5367
Paired distance between residences; M (SD)	16.48 (10.07)	15.94 (9.81)	0.0965

*IEHT* Inpatient-equivalent home treatment, *M* mean value, *SD* standard deviation, *SU* service user, *MW-Test* Wilcoxon-Mann-Whitney-Test

## Discussion

The results show that in the Brandenburg region we studied, SU generally were able to receive inpatient or inpatient-equivalent home treatment regardless of the travel time and distance to the hospital. It was not possible to prove a “Distance Decay” effect for either of the two settings or a particular primary diagnosis since the utilisation of health care services seems to increase with rising distance from the hospital rather than to diminish. Concerning the inpatient setting, the results confirm already existing evidence [3, 26].

Stulz, Hepp and colleagues argue that the usually more severe and acute clinical condition of SU treated in the inpatient setting, often taken to the hospital as an emergency or involuntary admission, reduces the influence of distance on the usage of the inpatient setting [3]. In contrast, the “Distance Decay” effect is clearly proven for community-based treatment offers, such as outpatient units or day care centres: SU that live further away from such a health care unit, utilise it to a lesser extent than SU with less travel time – regardless of the means of transport [1, 3]. This argument initially appeared equally plausible for the IEHT setting. It would follow that mainly SU who are easily reachable with the available means of transport, i.e. that live close to the hospital, would be treated with IEHT.

Yet the present results show that the implementation of IEHT is distributed in a more complex manner. SU in the observed region were more likely to be admitted to IEHT in those areas in which other SU living close by received the same treatment. This particularly applied to densely populated territories. To date, the increased rate of utilisation of health care services in urban sectors is well documented through the typically higher prevalence of psychiatric crises in comparison to rural districts [27–33].

Aside from this epidemiological factor, the decision whether a SU with a specific residence location could be treated with IEHT was also determined by institutional contingencies in the studied region. To facilitate admission of SU living further away from the IEHT team travel route, caseload and available staff had to be balanced so that the IEHT team could compensate for longer travel times [18].

### Implications for intensive home treatment in rural regions

The possibilities to offer spatially inclusive and comprehensive home treatment services in the examined region are restricted within the current framework of the IEHT. A conceivable scenario could be to dispatch IEHT teams from satellite sites spread throughout the catchment area instead of dispatching them from the headquarters of the hospital. This would presumably increase the range of the IEHT team. Alternatively, the caseload could be diminished to such an extent that even SU that live further apart could be treated by one and the same IEHT team. Yet there are strong limitations to the feasibility of this option, since from a caseload of roughly 4–5 downwards a cost-effective service provision would be impossible in the long term. This hypothesis is also supported by health economic evaluations, which provide ambiguous, or inconclusive, evidence of the cost-effectiveness of outreach care in rural regions [34].

To ensure the successful implementation of exhaustive intensive psychiatric outreach care in rural areas, other framework conditions than those of IEHT are necessary. Several different flexible outreach treatment models are already well established in the international scene [35], among these is worth mentioning the Flexible Assertive Community Treatment (= FACT) [36–38]. In a recent Danish study, FACT showed better effects and lower inpatient readmission rates than traditional community mental health care models [38]. Despite the possibility to flexibly control treatment intensity, in the Danish example FACT was offered at a high intensity, so that the caseload had to be reduced in rural versus urban study regions due to longer travel times [38].

Currently, a variety of integrated and outreach mental health service models exist in Germany, which allow for a flexible management of treatment intensity. For example, based on a global treatment budget (according to §64b social code V), it was possible to implement a form of mental health home treatment in very sparsely populated regions of Schleswig-Holstein in Northern Germany that covered the entire region through a flexible control of the treatment intensity [24, 25, 39]. A further similarly flexible alternative is the psychiatric acute home treatment at Bamberger Hof (close to Frankfurt, Germany), which is offered from the basis of a psychiatric outpatient centre [40]. Although these models are often not equivalent to inpatient treatment regarding treatment intensity, i.e. the frequency of contacts, they enable a basic outreach acute care that is less resource intensive than IEHT. By allowing for flexible treatment intensity in IEHT, for example through the elimination of the daily (in-person) treatment contacts requirement, a single care team could treat different SU on different days in varying parts of the catchment area.

A further possibility for increasing the flexibility of home treatment, and thus also for expanding its range of accessibility, would be to utilise digitalisation to reduce the number of in-person contacts with SU. In the context of the Covid-19 pandemic, the application of Video- and Telemedicine to reduce in-person contacts by intensive home treatment teams was internationally successfully tested [41]. A current systematic review concerning the digitalisation of IEHT describes, among others, the successful implementation of Blended-Care approaches, which allowed to offer IEHT in rural care service regions despite the restrictive legal framework [42–44]. In this way, the treatment could be provided digitally on specific days, given that it fits the clinical recommendation and the SU needs (and wish). In turn, the consequently saved travel time could for example be used for offering more (digital) contacts with the SU.

### Limitations and strengths

This is the first study that analyses the utilisation of intensive outreach versus inpatient psychiatric treatment from a geographical perspective based on routine hospital data. This study was limited to one particular region, which limits the generalisability of the results. A comparative analysis of more and different regions is thus still needed. Still, we were able to demonstrate a fundamental problem of outreach care services in larger catchment areas. The fact that the car travel route between hospital and place of residence diverges from the actual route of the IEHT team is considered a methodological limitation. The reason for this is that the SU in treatment - at least in the study region - are visited one after the other, which in many cases decreases the travel route per SU considerably. Yet the mean distance between hospital and place of residence as an indicator for the accessibility of a care service could still enable a basic comparison between the settings.

## Conclusions

The probability of a service user (= SU) to have access to and receive intensive home treatment is higher in densely populated regions of Germany than in areas with a lower population density or those located peripherally in catchment areas.

Our results suggest that the range of intensive outreach treatment does not depend on the distance/travel time of the treatment team to the residence of the SU but rather on its distance to the travel route of the home treatment team.

In order to present intensive outreach treatment as a truly equivalent alternative to inpatient psychiatric treatment, SU need to be able to access this form of treatment equally regardless of their place of residence within the catchment area. Making the restrictive legal framework of inpatient equivalent home treatment more flexible as well as supplementing Blended-Care approaches could contribute to increasing implementation of this concrete German form of intensive home treatment even in rural regions.

## Data Availability

The datasets generated and/or analysed during the current study are not publicly available due to privacy restrictions but are available from the corresponding author on reasonable request.

## Abbreviations

FACT: Flexible Assertive Community Treatment
GIS: Geographic Information System
IEHT: Inpatient-Equivalent Home Treatment
IHR: Immanuel Hospital Rüdersdorf
SU: Service User
